# Editorial: Application of multi-omics technologies to explore novel biological process and molecular function in immunology and oncology

**DOI:** 10.3389/fgene.2024.1403796

**Published:** 2024-04-25

**Authors:** Xinmin Li, Ye Wang, Jihong Yang, Anton Buzdin

**Affiliations:** ^1^ The Technology Center for Genomics and Bioinformatics, University of California, Los Angeles, CA, United States; ^2^ Qingdao University, The Affiliated Qingdao Central Hospital of Qingdao University, Qingdao, China; ^3^ College of Pharmaceutical Sciences, Zhejiang University, Hangzhou, China; ^4^ BoYu Intelligent Health Innovation Laboratory, Hangzhou, China; ^5^ World-Class Research Center “Digital Biodesign and Personalized Healthcare”, Sechenov First Moscow State Medical University, Moscow, Russia; ^6^ Moscow Center for Advanced Studies, Moscow, Russia; ^7^ Shemyakin-Ovchinnikov Institute of Bioorganic Chemistry, Moscow, Russia

**Keywords:** genomics, transcriptomics, proteomics, metabolomics, interactomics, cancer treatment, molecular diagnostics

Multiomics profiling offers the advantage of providing additional dimensions to biomedical studies (Wang et al., 2023). Being focused primarily on the quantitative and qualitative assessment of different levels of gene regulation, Multiomics can bring researchers to a next level of integration of functional data including genomic, epigenetic, transcriptomic, proteomic, and metabolomic profiles (Buzdin et al., 2019). This makes it possible to perform high throughput in-depth investigations of molecular mechanisms leading to carcinogenesis in individual cases or to generalize the results on the level of bigger patient cohorts or specific tumor molecular classes (Bonetti et al., 2023; Thiery and Fahrner, 2023). One of the most important aspects here deals with the immunity-related mechanisms of tumor growth and cancer therapy (Wang et al., 2021). In this Research Topic, attention was paid to the integration of Multiomics analyses in molecular cancer research. In this respect, construction of computational bioinformatic models aggregating various dimensions of experimental data is vital for successful annotation and comprehension of the wealth of Multiomics profiles (Buzdin A et al., 2021). This approach offers insights into complex regulatory mechanisms underlying emergence, growth, metastasis, and heterogeneity of various cancer types (Nobre et al, 2022; Rozenberg et al, 2023; Zhong et al, 2023). It can also pave the way for further exploring molecular complexity of cancers and offering new strategies for more efficient and/or personalized therapy.

Lung cancers lead worldwide as the cause of oncology-related deaths. They are highly heterogeneous in their cytologic and molecular phenotypes, and the advancements in Multiomics methodologies hold a promise to better understand and personalize treatment of these tumors. Using Multiomics-based regulatory networks, Díaz-Campos et al. constructed robust computational models elucidating omics data interconnectedness in lung cancer and enabling systematic generation of mechanistic hypotheses. The authors proposed a semi-automated theoretical and computational framework for generating network models depicting regulatory constraints on biological functions. In the molecular profiles of lung adenocarcinoma and squamous cell carcinoma, this approach could successfully identify enriched functions and novel regulatory characteristics in analyzed omics data, focusing on methylation of CpG islets and expression of protein-coding genes and micro RNAs.

Another aspect of high throughput molecular lung cancer research was raised by Lin et al. who compared gene expression profiles of 3,243 lung adenocarcinoma patients extracted from 22 annotated published datasets. They found statistically significant associations between the expression of senescence-related genes with advanced tumor stage and pathological grade, as well as with shorter survival characteristics. In addition, expression of senescence-related genes in the tumor microenvironment was connected with worse response on checkpoint immunotherapy, and better response on chemotherapy, thus suggesting their putative diagnostic value as the predictive biomarkers.

In oral squamous cell carcinoma, overall loss of 5-methylcytosine and 5-hydroxymethylcytosine have been previously observed. However, no simultaneous genome-wide mapping of such modifications in oral cancers was previously accomplished. Using parallel whole-genome bisulfite sequencing and whole-genome oxidative bisulfite sequencing, Zhao et al. for the first time managed to characterize 5-methylcytosine and 5-hydroxymethylcytosine profiles with a genome-wide and single-base-resolution in paired primary oral squamous cell carcinoma samples and their normal adjacent tissues. In oral squamous cell carcinoma, a total of 6,921 differentially methylated regions and 1,024 differentially hydroxymethylated regions were identified in the promoters of known genes. Compared to bidirectional modification with 5-methylcytosine and 5-hydroxymethylcytosine, unidirectional modification of promoters was found to be associated with a bigger fold-change of the gene expression. Furthermore, genes bearing unidirectional modification appeared to be enriched in molecular pathways promoting cell proliferation and activation of receptor tyrosine kinase signaling. These results suggest a functional impact for this previously unexplored mode of gene expression regulation in oral carcinomas.

Finally, Xiao et al. reported a rare subtype of diffuse large B-cell lymphoma that was accompanied by an enhanced level of monoclonal immunoglobulin M (IgM) paraprotein in serum. Following treatment, the monoclonal IgM disappeared in most of these patients, but one 59-year-old male patient showed continuously remarkably elevated IgM levels after therapy. The patient was diagnosed with GCB subtype per Hans algorithm, stage IA with involvement of the right cervical lymph node. After six cycles of combined immune checkpoint therapy and chemotherapy, complete metabolic remission was achieved, but an elevated level of serum IgM persisted. To investigate this case in more detail, pathologic, immunophenotypic, and molecular analyses of lymph node and bone marrow samples were performed pre- and post-treatment. The authors observed bone marrow infiltration with lymphoplasmacytic cells, and detected by flow cytometry immunophenotypic profile characteristic for the diagnosis of Waldenström macroglobulinemia. Further next-generation sequencing of the lymph node biosample confirmed the initial diagnosis by finding co-occurring point mutations in *MYD88L265P* and *CD79B* genes. Additionally, two different dominant clonotypes of the immunoglobulin heavy chain were detected in the lymph node and bone marrow by sequencing, which suggests two independent clonal origins for this tumor case. This case contributes to understanding the origin and biological characteristics of diffuse large B-cell lymphoma co-occurring with Waldenström macroglobulinemia.
